# Influence of interradicular and palatal placement of orthodontic mini-implants on the success (survival) rate

**DOI:** 10.1186/s13005-017-0147-z

**Published:** 2017-06-14

**Authors:** Jan Hourfar, Dirk Bister, Georgios Kanavakis, Jörg Alexander Lisson, Björn Ludwig

**Affiliations:** 1 Department of Orthodontics, Saarland University, Homburg, Germany; 2 Department of Orthodontics, Guy’s and St Thomas’ NHS Foundation Trust and King’s College Dental Institute, London, UK; 3 Department of Orthodontics, Tufts University School of Dental Medicine, Boston, USA; 4 Private Practice, Am Bahnhof 54, 56841 Traben-Trarbach, Germany

## Abstract

**Background:**

The purpose of this retrospective cohort study was to investigate the success rates of orthodontic mini-implants (OMIs) placed in different insertion sites and to analyse patient and site- related factors that influence mini-implant survival.

**Methods:**

Three hundred eighty-seven OMIs were inserted in 239 patients for orthodontic anchorage and were loaded with a force greater than 2 N. Two different insertion sites were compared: 1. buccal inter-radicular and 2. palatal, at the level of the third palatal ruga. Survival was analysed for location and select patient parameters (age, gender and oral hygiene). The level of statistical significance was set at *p* < 0.05.

**Results:**

The overall success rate was 89.1%. There were statistically significant differences between insertion sites; success rate was 98.4% for OMIs placed in the anterior palate and 71% for OMIs inserted buccal between roots (*p* < 0.001).

**Conclusions:**

Success rate of OMIs was primarily affected by the insertion site. The anterior palate was a more successful location compared to buccal alveolar bone.

## Background

The introduction of temporary anchorage devices (TADs) for skeletal anchorage in orthodontics promised to improve biomechanical possibilities for tooth movement [1, 2]; orthodontic mini-implants (OMIs) are the smallest TADs available [3]. Due to their reduced size, OMIs can be inserted at various sites in both jaws [4].

OMIs are generally well accepted by patients [5]; they offer affordable support for anchorage demanding orthodontic biomechanics [4]. Other TADs such as orthodontic mini-plates need soft tissue surgery and are comparatively complex to insert and use; insertion is usually undertaken by oral surgeons. OMIs however can be relatively easily placed and removed, causing little discomfort to the patient [6]; this can be undertaken by the orthodontist during a routine visit [7].

A large body of evidence on success rates [6, 8–40] of OMIs exists, showing an average of approximately 84% [8, 9]. However, considerable variation between different anatomical insertion sites has been reported [41]. A meta-analysis of 52 studies on OMIs reported an overall failure rate of 13.5% [42].

Parasagittal insertion of OMIs in the anterior palate has one of the highest success rates [4]. A prospective study of Straumann® palatal implants revealed success rates of 95.7% [43]. A systematic review [9] showed that palatal implants have a better success rate compared to inter-radicular OMIs. The Straumann® palatal implant has a surface area of about 54 mm^2^ whilst two joined OMIs feature a larger combined surface area (2 × 45.34 mm^2^) [44]. The success of OMIs may hence be correlated to an increased implant-to-bone contact area rather than the properties of the screws themselves or other proposed factors.

A number of orthodontic appliances, (e.g. for rapid palatal expansion (RPE) [45] or maxillary molar distalization [46]) utilise two OMIs that are usually inserted parasagittal in the anterior palate. Parasagittal insertion is mandatory for OMI-supported RPE, whereas various designs have been described for distalization of molars in the upper jaw, some of which use inter-radicular insertion sites [47].

Success rates greater than 80% [8, 9] should encourage orthodontists to use OMIs. Knowledge of insertion sites with high success rates is therefore crucial as it will affect clinical decision making. Success rates of two joined palatal OMIs have not yet been compared to success rates of appliances supported by inter-radicular insertion sites and to our knowledge no conclusive data on this subject is currently available.

The purpose of this retrospective cohort study was to investigate the success rates of orthodontic mini-implants (OMIs) placed either on the buccal side between roots of the teeth or palatally at the level of the third palatal ruga, and also to determine patient related factors that may have an impact on success rates.

## Methods

### Study sample

All OMIs had identical dimensions (diameter 1.7 mm; length 8 mm), and were manufactured by the same company (OrthoEasy®, Forestadent, Pforzheim, Germany). Other inclusion criteria were: complete patient records including panoramic and cephalometric radiographs, intraoral photographs of applied orthodontic biomechanics and oral hygiene index measurements (Approximal Plaque Index (API) score) at every visit.

After application of the inclusion criteria, the search generated 239 patient records that were eligible for this investigation (137 females and 102 males). The median age of the patients was 13.8 years (interquartile range (IQR) 11.0–16.9 years). A total of 387 OMIs were inserted: 190 in the anterior palate and 197 in buccal inter-radicular sites.

### Methodology

**Fig. 1:**
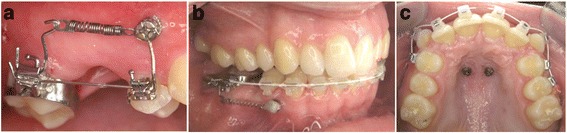
Vestibular inter - radicular insertion was at the height of the muco-gingival line in the maxilla (**a**) and the mandible (**b**). **c** Palatal orthodontic mini-implants were place only in the anterior palate, directly posterior to the third palatal rugae

All OMIs were inserted in a private orthodontic practice over a three years observation period. This implant system has an anodized surface, features a self-tapping and self-drilling design and is made from titanium-vanadium alloy (Ti-6Al-4 V). Following patient consultation and written consent, 0.2 ml to 0.5 ml of local anaesthetic were used (Ultracain® D-S, Sanofi-Aventis Deutschland GmbH, Frankfurt, Germany), and OMIs were inserted without soft tissue incision or predrilling. The OMIs were inserted at two different sites:

1. Buccal in either upper or lower jaw, using the cortical bone between the roots at the height of the mucogingival line, (Fig. 1a and b) [48].
2. Palatal immediately posterior to the third palatal rugae (Fig. 1c) [49, 50]. Palatal insertion always included the use of two OMIs that were connected by the orthodontic appliance.

**Fig. 2:**
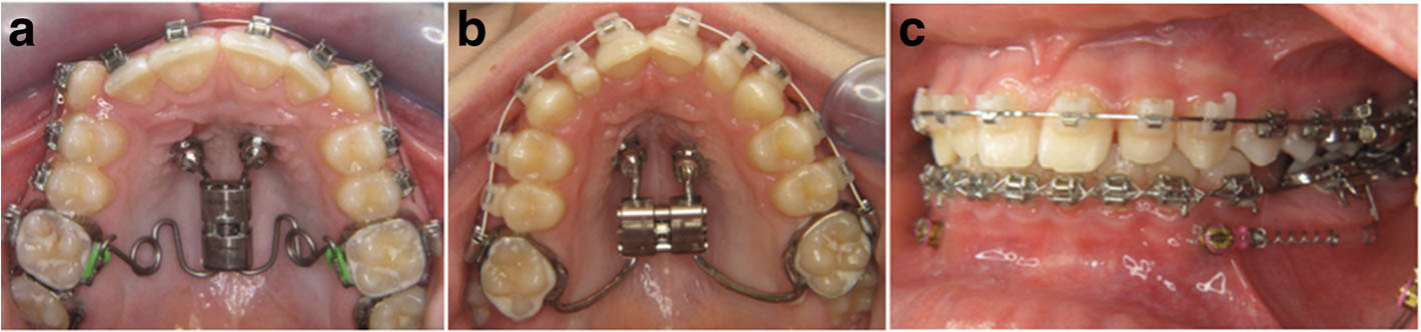
**a** Distalization of posterior teeth utilizing palatal orthodontic mini-implants **b** Rapid palatal expansion with hybrid RPE, anchored on two palatal orthodontic mini-implants. **c** Space closure utilising vestibular orthodontic mini-implants

All buccal OMIs were loaded on the day of insertion. The typical use was molar protraction with a force >2 N, using standardized Nickel-Titanium (NiTi) coil springs (Fig. 1a and b, Fig. 2c).

Palatal OMIs were loaded within 3 days after placement as the attached appliance had to be manufactured in a dental laboratory. All OMIs were used for direct anchorage. All biomechanics applied to the OMIs produced a force of >2 N and all palatal appliances worked bilaterally therefore exerting equal forces to both implants.

The palatal OMIs used in this study were either for maxillary molar distalization [46] (Fig. 2a), or rapid palatal expansion using a hybrid RPE (“hybrid hyrax”, Wilmes et al. [45]) (Fig. 2b). Both of these appliances were directly attached to the OMIs and exerted equal forces of (>2 N) per implant; the exact force values have been previously investigated elsewhere [46, 51].

OMIs remaining in situ over the entire period of treatment that required anchorage were recorded as successful. Premature loss or if removal of the OMI became necessary before achieving the defined treatment aims were charted unsuccessful.

#### Data collection and statistical analysis

All data were tabulated in a Microsoft Excel® 2007 file (Microsoft Corp., Richmond, Wash., USA). SPSS® for Windows version 22.0 (IBM Corp., Armonk, NY, USA) was used for all statistical analyses. The following parameters were analysed in relation to OMI success rates: 1) insertion site (anatomy); and 2) patient demographic data (age, gender and oral hygiene). Normal distribution of metrical parameters was assessed using Kolmogorov-Smirnov (K-S) tests. Comparisons between nominal variables were performed with the Pearson’s chi-squared test or Fisher’s exact test for non-parametric data. Continuous data not following a normal distribution were analysed using Mann-Whitney U test and descriptive statistics; median and interquartile range (IQR) are displayed accordingly. Survival rates were calculated with the Kaplan-Meier estimator, and their significance was evaluated with log rank tests. Additional Cox regression analysis of multiple variables was performed. Statistical significance was set at *p* < 0.05.

## Results

Three hundred twenty-eight out of 387 orthodontic mini-implants were considered successful; the overall success rate was 84.8% over the observation period.

### Analysis by anatomical site

Significant differences in success rates were observed between the palatal and the inter-radicular insertion site (Table 1). The success rate of palatal OMIs was 98.9%. Only 2 out of 190 palatal OMIs were lost. Those were inserted in the same patient, providing anchorage for molar distalization; The implants had to be removed before the treatment objectives had been achieved because they were loose.

**Table 1:** Success rates by insertion site

Insertion site	n	success rate		*p*-value
		%	absolute numbers	
anterior palate	190	98.9	188	< 0.001
inter-radicular	197	71.1	140	
total	387	84.8	328	

Interradicular OMIs were successful in 71.1% of the cases. There was no statistically significant difference in success rates between maxillary inter-radicular and mandibular inter-radicular OMIs (*p* = 0.628).

### Analysis by patient factors

Patient parameters that were assessed included, age and gender (Table 2) and these parameters had an impact on success rates. OMIs inserted in patients older than 30 years were found to have a 29.5% failure rate compared to those used in younger patients that showed lower failure rates of 14.8% (20–30 years) and 13.3% (6–20 years). However, this difference was statistically significant only for the youngest group (6–20 years). A significant difference in success rates was also noted between male and female patients.

**Table 2:** Relationship of success rates of OMIs to oral hygiene, age and gender

patient factor		n (total)	success rate		*p*-value
			%	n	
oral hygiene	good (API < 30%)	352	84.9	299	0.743
	poor (API > 30%)	35	82.9	29	
		387		328	
age	6–20 years old	316	86.7	274	0.002**
	20–30 years old	27	85.2	23	0.287
	> 30 years old	44	70.5	31	0.494
		387		328	
gender	male	160	80.0	128	0.029*
	female	227	88.1	200	
		387		328	

*p < 0.05; **p < 0.01

### Survival rate analysis

**Fig. 3:**
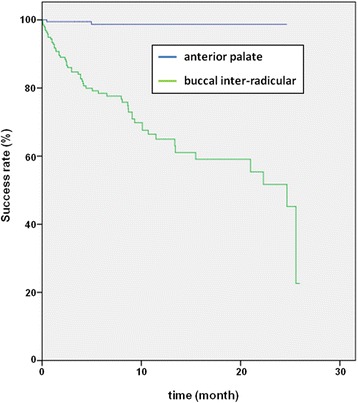
Survival rates based on Kaplan-Meier estimates were calculated between day of insertion until loss, premature removal or scheduled removal of orthodontic mini-implants

Survival rates based on Kaplan-Meier estimates were calculated from day of insertion until day of implant loss, early or scheduled removal of mini-implants. 59 OMIs were either lost or removed prematurely. Analysis of survival rates for the anterior palate compared to inter-radicular insertion sites demonstrated a better performance of palatal OMIs (Fig. 3). Those remained in place for 24.4 months on average. Interradicular insertion showed higher loss rates in the first 13 months with an average survival of 17.4 months. The correlation between insertion site and the failure rate of OMIs was statistically significant (*p* < 0.001). The results of the Cox-Regression are displayed in Table 3.

**Table 3:** Parameters according to Cox regression analysis

variable	hazard ratio	95% CI	p
insertion site	0.036	0.009–0.150	<0.001
age	1.000	0.977–1.023	0.970
gender	1.565	0.928–2.640	0.093
oral hygiene	2.227	0.931–5.329	0.072

*CI* confidence interval

## Discussion

During the last decade, numerous studies have assessed the success rates of orthodontic mini-implants [6, 8–11, 14, 16, 17, 19, 20, 22–28, 31, 32, 36, 38, 39] and reported values ranging between 66% and 100% [41]. Results from these investigations reveal a mean success rate of 85.50% (median value 85.46%), which is very close to the results (83.6%) of two systematic reviews [8, 9]. The success rate of our retrospective cohort study (84.8%) is similar.

Approximately half (50.9%) of all OMIs in this study were inserted in the anterior palate, and 98.9% of those were successful. However, palatal OMIs were always inserted and used in pairs. Connecting two OMIs may be the reason for better stability due to the increased surface area of two mini-implants [52]. Two palatal OMIs provide a nearly identical surface area as one standard size palatal implant. The failure rate of the latter has been reported to be between 4.3% and 9% [43, 53–55]. Only in one patient in our cohort both palatal OMIs were lost; these were providing skeletal anchorage for a distalization appliance.

Orthodontic mini-implants inserted in the buccal alveolus were successful in 71.1% of all cases and similar values have also been reported by other investigators [10, 11, 17, 24, 38, 39].

Success rates were significantly influenced by the combination of load and insertion site. In our investigation palatal OMIs were nearly always successful. Buccal mini-implants were clearly less successful. 59 (15.3%) out of 387 inserted OMIs were lost, and 57 of those had been inserted buccal between roots to support space closure mechanics. It appears that the combination of inter-radicular insertion combined with the type of use resulted in a poorer survival rate.

Existing evidence suggests that moderate loading of OMIs is preferable [56, 57]. Osseointegrated dental implants, featuring treated surfaces, larger diameters and lengths, are probably better suited for the application of heavy loads [58]. Bicortical insertion of OMIs has also been recommended for improved stability when heavy forces are applied [59, 60].

Anchorage design might have an effect on success rates. The findings of Antoszewska et al. [10] indicated that indirect use seemed to have a higher success rate (96.96%) than direct loading (92.60%) but this difference was not statistically significant. In our study, all OMIs were directly loaded, which might have influenced the success rate for buccal inter-radicular insertion (71.1%). In addition all directly loaded OMIs in the palate were joint to one appliance, while the buccal inter-radicular OMIs were used individually and this may have had an impact on success rates.

Other than oral hygiene, gender and age revealed statistically significant differences, but only for younger patients (6–20 years). It is remarkable that OMIs inserted in patients older than 30 years had a failure rate that was twice as high (29.5%) compared to those used in younger patients: 14.8% (20–30 years) and 13.3% (6–20 years), respectively. These differences might be explained by the fact that OMIs in the older patient group (>30 years) were mostly used for molar protraction with forces >2 N, with an associated success rate of 70.5%.

Throughout the entire study period, 59 of 387 OMIs were lost or removed prematurely with an average survival time of 24.4 months. OMIs inserted between roots demonstrated considerably higher loss rates within the first 5 months, with an average survival of 17.4 months. Consequently, the correlation between insertion site and loss of OMIs was highly significant (*p* < 0.001), confirming results from previous research [10, 39].

In summary, our investigation was a large retrospective cohort study and our results concur with previous research [24, 61]. Apart from location and application of OMI we also investigated selected patient related factors. To our knowledge this is the first time that success rates of two joined parasagittal palatal OMIs were compared to inter-radicular insertion sites, adding new data supporting the exiting body of evidence. Maybe not surprisingly the success rate of palatal OMIs of our study was very similar to that of the Straumann® palatal implant [43]. This suggest that two joined palatal OMIs might be used as an alternative for a palatal implant; OMIs can be loaded immediately compared to palatal implants that require 12 weeks’ for osseointegration. However once osseointegrated, palatal implants remain absolutely stable when loaded compared to OMIs [62, 63]. A drawback of our investigation is the retrospective design which may have introduced bias and prospective data from a randomized trials may make results more reliable [64].

Extensive research has been undertaken [42, 65] trying to analyse risk factors for OMI failure but many questions remained unanswered [42]. Further research, ideally using prospective randomized designs or a prospective cohort investigation in this field are needed.

## Conclusions

This retrospective investigation demonstrated that the success rate of OMIs loaded with forces greater than 2 N was mainly affected by the site of their insertion. Two OMIs inserted in the anterior palate, joined together and used for direct anchorage offered survival rates close to 100%. Individually used OMIs inserted between roots in the buccal alveolus resulted in significantly lower (71.1%) success rates; there was no statistically significant difference for upper and lower buccal insertion sites.
